# The Computation of Complex Dispersion and Properties of Evanescent Lamb Wave in Functionally Graded Piezoelectric-Piezomagnetic Plates

**DOI:** 10.3390/ma11071186

**Published:** 2018-07-10

**Authors:** Xiaoming Zhang, Zhi Li, Jiangong Yu

**Affiliations:** School of Mechanical and Power Engineering, Henan Polytechnic University, Jiaozuo 454000, China; zxmworld11@hpu.edu.cn (X.Z.); jixielizhi@126.com (Z.L.)

**Keywords:** evanescent wave, polynomial approach, functionally graded piezoelectric-piezomagnetic material, dispersion, attenuation

## Abstract

Functionally graded piezoelectric-piezomagnetic material (FGPPM), with a gradual variation of the material properties in the desired direction(s), can improve the conversion of energy among mechanical, electric, and magnetic fields. Full dispersion relations and wave mode shapes are vital to understanding dynamic behaviors of structures made of FGPPM. In this paper, an analytic method based on polynomial expansions is proposed to investigate the complex-valued dispersion and the evanescent Lamb wave in FGPPM plates. Comparisons with other related studies are conducted to validate the correctness of the presented method. Characteristics of the guided wave, including propagating modes and evanescent modes, in various FGPPM plates are studied, and three-dimensional full dispersion and attenuation curves are plotted to gain a deeper insight into the nature of the evanescent wave. The influences of the gradient variation on the dispersion and the magneto-electromechanical coupling factor are illustrated. The displacement amplitude and electric potential and magnetic potential distributions are also discussed in detail. The obtained numerical results could be useful to design and optimize different sensors and transducers made of smart piezoelectric and piezomagnetic materials with high performance by adjusting the gradient property.

## 1. Introduction

Due to the excellent coupling behavior among mechanical, electric, and magnetic fields, piezoelectric-piezomagnetic composites (or magneto-electro-elastic material) composed of piezoelectric and piezomagnetic phases have been increasingly applied to different engineering structures, especially to the smart or intelligent systems as intelligent sensors, damage detectors, etc. [1]. It is found that the smart structures made of functionally graded materials (FGM) possess a better structural performance than traditional composite materials. The concept of FGM has been extended to the development of new piezoelectric-piezomagnetic materials appointed functionally graded piezoelectric-piezomagnetic materials, which can realize the smooth transition of the physical constitutive parameters of the piezoelectric and piezomagnetic materials. FGPPMs have been used in some devices to improve their efficiency and other features. Many applications are closely connected with the vibration and wave propagation of FGM and FGPPM [2,3,4,5]. Dispersion relations and wave mode shapes are very important for understanding dynamic behaviors of structures. Wave propagation features in FGPPM plates could be also useful in designing and optimizing the high-accuracy sensors and transducers [6,7].

With the remarkable achievements in fabrication of FGPPM during the decades, many investigators have turned attention to the study of wave propagation in such materials. Wang and Rokhlin [8] presented the differential equations governing the transfer and stiffness matrices for a functionally graded generally anisotropic magneto-electro-elastic medium and calculated the surface wave velocity dispersion. Pan et al. [9] derived an exact solution for the multilayered plate made of functionally graded, anisotropic and linear magneto-electro-elastic materials based on Pseudo-Stroh formalism. Bhangale et al. [10] carried out the free vibration studies on the simply supported functionally graded magneto-electro-elastic plate by semi-analytical finite element method. Wu et al. [11] investigated the wave propagating characteristics in the non-homogeneous magneto-electro-elastic plates by using orthogonal polynomial approach. By employing the power series technique, Cao [12] investigated the Lamb wave propagation in FGPPM plates. Singh and Rokne [13] investigated the SH wave propagating in FGPPM structures. Xiao et al. [14] investigated the dispersion properties of wave propagation in the functionally graded magneto-electro-elastic plate by the Chebyshev spectral element method.

As is reviewed above, so far, studies on the guided wave in FGPPM structures are limited to the propagating waves, but the evanescent waves have not been investigated. Recently, some studies on pseudo surface acoustic waves (PSAW) in piezoelectric half-spaces find that the PSAW modes have higher velocities and lower attenuations, compared to the classical surface acoustic waves [15,16]. Such modes make the piezoelectric device possess higher resolution. Evanescent wave modes also have the similar features. According to the classification of Auld [17], the complete wave modes consist of propagating modes with real wave number and evanescent modes with complex or purely imaginary wave number. Note that evanescent modes represent local modes that would exist at discontinuities and decay with propagating distance (so referred to as evanescent or non-propagating wave). As early as 1955, Lyon [18] obtained the purely imaginary roots of the dispersion equation for an elastic plate. Remarkable is the work done by Mindlin who demonstrated the presence of complex roots of the Rayleigh-Lamb equation [19]. Freedman [20] studied the imaginary valued Lamb mode spectra covering virtually the full range of the Poisson ratio. Quintanilla et al. [21] calculated the full spectrum for guided wave problems in plates and layered cylinders using a spectral collocation method. More recently, Yan and Yuan [22,23] discussed the potential application of evanescent waves in structural health monitoring and investigated the conversion of evanescent SH and Lamb waves into propagating waves using a semi-analytical approach. Chen et al. [24] studied theoretically the real-valued and imaginary-valued SH waves in a piezoelectric plate of cubic crystals. These researches focused on the simple material and purely imaginary modes. In fact, the search of complex roots corresponding to evanescent waves is a difficult task for FGM with material properties of variable coefficients. To the best of the authors’ knowledge, the evanescent waves in FGM or piezoelectric-piezomagnetic composite have not been studied before, which is the motivation of this study.

In this paper, an analytic method based on polynomial expansions is proposed to calculate guided waves in FGPPM plates. The presented method can replace the problem of computing a transcendental dispersion equation by a general eigenvalue problem in wave number. The complete solutions of the dispersion equation, including the purely real, purely imaginary and complex solutions, can be obtained. We plot the full dispersion curves in three dimensional (3D) frequency-complex wave number space to gain a better and deeper insight into the characteristics of evanescent waves. Two known cases are given to validate this approach. The characteristics of evanescent guided waves in various FGPPM plates are illustrated. The effects of different graded fields on the dispersion curves and the coupled electromechanical factor are investigated. The displacement amplitude and electric potential and magnetic potential distributions are also discussed in detail.

## 2. Mathematics and Formulation of the Problem

**Figure 1:**
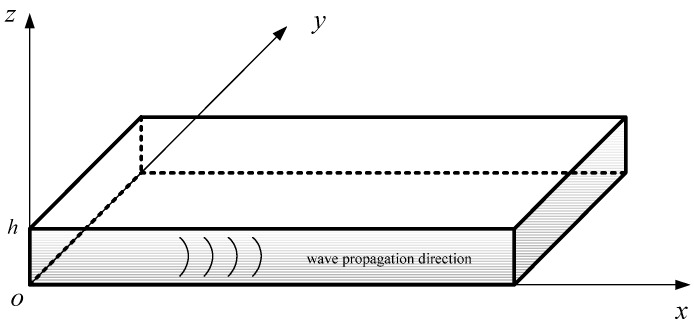
Geometry of the problem.

Consider a FGPPM plate with varying material properties with regard to thickness (the *z*-axis). The plate described in Cartesian coordinate system (*x*, *y*, *z*), is infinite horizontally but finite in the *z* direction with a thickness *h*, occupies the region 0 ≤ *z* ≤ *h*, as shown in Figure 1. The wave propagates along the *x* direction, and the upper and bottom surface of the plate are traction free.

For the piezoelectric-piezomagnetic medium, the governing field equations (Equation (1)) and the generalized constitutive relations (Equation (2)) can be expressed as [12]:

(1) $$\sigma_{ij,j}=\rho {\ddot{u}}_{i},\;D_{i,i}=0,\;B_{i,i}=0$$

(2) $$\sigma_{ij}=C_{ijkl}\varepsilon_{kl}-e_{kij}E_{k}-q_{ijk}H_{k},\;D_{i}=e_{ikl}\varepsilon_{kl}+\in_{ik}E_{k}+g_{ik}H_{k},\;B_{i}=q_{ijk}\varepsilon_{jk}+g_{ik}E_{k}+\mu_{ik}H_{k}$$

(3) $$\varepsilon_{ij}=\frac{1}{2}\left(u_{i,j}+u_{j,i}\right),\;E_{i}=-\varphi_{,i},\;H_{i}=-\psi_{,i}$$

Generalized geometric equations under a rectangular coordinates system are in the above Equations (1)–(3), $\sigma_{ij}$ is the stress tensor, $D_{i}$ is the electric displacement and $B_{i}$ is the magnetic induction $C_{ijkl},\;e_{kij},\;q_{ijk},\;\in_{ik},\;g_{ik}$ and $\mu_{ik}$ are the elastic, piezoelectric, piezomagnetic, dielectric, magnetic, and magnetoelectric parameters of the FGPPM, respectively, while all of them, including the mass density *ρ*, are functions of *z*. According to Einstein summation convention, where *i*, *j*, *k* and *l* = 1, 2, 3 corresponding to *x*, *y*, *z* directions, respectively. $\varepsilon_{kl},\;E_{k}$ and $H_{k}$ are the strain tensor, the electric field, and magnetic field, respectively. *u_i_* (*i* = *x*, *y*, *z*) denotes the mechanical displacement component in the *i*th direction. *φ* and *ψ* are the electric potential and magnetic potential. Comma in subscripts and superposed dot denote spatial and time derivatives, respectively.

For Lamb waves propagating along the *x* direction, the displacement components, electric potential and magnetic potential can be expressed as

(4) $$u_{i}(x,z,t)=\exp(ikx-i\omega t)U_{i}(z),\;\varphi (x,z,t)=\exp(ikx-i\omega t)X(z),\psi (x,z,t)=\exp(ikx-i\omega t)Y(z)$$

where *U_i_* (*i* = *x*, *z*) represents the amplitude of the displacements in the *i*th directions, *X* and *Y* represent the amplitude of electric potential and magnetic potential, respectively. *k* is the wave number, *ω* is the angular frequency, and *i* is the imaginary number.

Since the material properties change gradually with thickness and are the functions of *z*, they can be fitted into the following form:

(5) $$f(z)=f^{\left(l\right)}{\left(z/h\right)}^{l},l=0,1,2\ldots ,L$$

where *f* (*f* = *ρ*, *C*, *e*, *∈*, *q*, *g* and *μ*) denotes material parameters, *l* is the order number, *f*^(*l*)^ is the coefficient. For homogeneous material, $f(z)=C^{\left(0\right)}$, and when *l* > 0, *f*(*l*) is zero.

The following boundary and continuous conditions should be satisfied as follows. For the traction-free boundary condition, it requires that $\sigma_{zz}{|}_{z=0,h}=0$, $\sigma_{xz}{|}_{z=0,h}=0$, $\sigma_{yz}{|}_{z=0,h}=0$. For electric and magnetic open circuit, $D_{z}{|}_{z=0,h}=0$, $B_{z}{|}_{z=0,h}=0$, and for electric and magnetic shorted circuit, $\varphi {|}_{z=0,h}=0$, $\psi {|}_{z=0,h}=0$. 

Then take the traction-free and electrical and magnetic open-circuit boundary conditions as an example. Considering the boundary of material, the position-dependent material parameters are given by:

(6) f(z)=f(z)π(z)

where *π*(*z*) is a rectangular window function defined by $\pi (z)=\left\{ \begin{array}{l} \begin{array}{cc} 1, & 0\leq z\leq h \end{array}\\ \begin{array}{cc} 0, & elsewhere \end{array} \end{array}\right.$, whose derivative is a Dirac’s delta function, δ(z−h)−δ(z). Then the boundary conditions can be automatically incorporated in the constitutive relations [25].

To reduce the number of resolving equations, we substitute Equations (3)–(6) into Equation (2) with following substitution into Equation (1). Consequently, the governing differential equations in terms of the displacement, electric potential and magnetic potential components can be obtained. Here, the case of an orthotropic FGPPM plate with the *z* direction polarization is given:

(7a) $$\begin{array}{l} {\left(\frac{z}{h}\right)}^{l}[C_{55}^{\left(l\right)}U^{\prime \prime }+lz^{-1}C_{55}^{\left(l\right)}U^{\prime }+ik\left(C_{13}^{\left(l\right)}+C_{55}^{\left(l\right)}\right)W^{\prime }+ik\left(e_{15}^{\left(l\right)}+e_{31}^{\left(l\right)}\right)X^{\prime }+ik\left(q_{15}^{\left(l\right)}+q_{31}^{\left(l\right)}\right)Y^{\prime }\\ -k^{2}C_{11}^{\left(l\right)}U+likz^{-1}\left(C_{55}^{\left(l\right)}W+e_{15}^{\left(l\right)}X+q_{15}^{\left(l\right)}Y\right)]\pi \left(z\right)+\left(\delta (z-0)-\delta (z-h)\right){\left(\frac{z}{h}\right)}^{l}(C_{55}^{\left(l\right)}U^{\prime }\\ +ikC_{55}^{\left(l\right)}W+ike_{15}^{\left(l\right)}X+ikq_{15}^{\left(l\right)}Y)=-\frac{\rho^{\left(l\right)}z^{l}\omega^{2}}{h^{l}}U\pi \left(z\right) \end{array}$$

(7b) $$\begin{array}{l} {\left(\frac{z}{h}\right)}^{l}[C_{33}^{\left(l\right)}W^{\prime \prime }+e_{33}^{\left(l\right)}X^{\prime \prime }+q_{33}^{\left(l\right)}Y^{\prime \prime }+ik\left(C_{13}^{\left(l\right)}+C_{55}^{\left(l\right)}\right)U^{\prime }+lz^{-1}\left(C_{33}^{\left(l\right)}W^{\prime }+e_{33}^{\left(l\right)}X^{\prime }+q_{33}^{\left(l\right)}Y^{\prime }\right)\\ +likz^{-1}C_{13}^{\left(l\right)}U-k^{2}\left(C_{55}^{\left(l\right)}W+e_{15}^{\left(l\right)}X+q_{15}^{\left(l\right)}Y\right)]\pi \left(z\right)+\left(\delta (z-0)-\delta (z-h)\right){\left(\frac{z}{h}\right)}^{l}(C_{33}^{\left(l\right)}W^{\prime }\\ +e_{33}^{\left(l\right)}X^{\prime }+q_{33}^{\left(l\right)}Y^{\prime }+ikC_{13}^{\left(l\right)}U)=-\frac{\rho^{\left(l\right)}z^{l}\omega^{2}}{h^{l}}W\pi \left(z\right) \end{array}$$

(7c) $$\begin{array}{l} {\left(\frac{z}{h}\right)}^{l}[e_{33}^{\left(l\right)}W^{\prime \prime }-\in_{33}^{\left(l\right)}X^{\prime \prime }-g_{33}^{\left(l\right)}Y^{\prime \prime }+ik\left(e_{15}^{\left(l\right)}+e_{31}^{\left(l\right)}\right)U^{\prime }+lz^{-1}\left(e_{33}^{\left(l\right)}W^{\prime }-\in_{33}^{\left(l\right)}X^{\prime }-g_{33}^{\left(l\right)}Y^{\prime }\right)\\ +likz^{-1}e_{31}^{\left(l\right)}U-k^{2}e_{15}^{\left(l\right)}W+k^{2}\in_{11}^{\left(l\right)}X+k^{2}g_{11}^{\left(l\right)}Y]\pi \left(z\right)\\ +\left(\delta (z-0)-\delta (z-h)\right){\left(\frac{z}{h}\right)}^{l}(e_{33}^{\left(l\right)}W^{\prime }-\in_{33}^{\left(l\right)}X^{\prime }-g_{33}^{\left(l\right)}Y^{\prime }+ike_{31}^{\left(l\right)}U)=0 \end{array}$$

(7d) $$\begin{array}{l} {\left(\frac{z}{h}\right)}^{l}[q_{33}^{\left(l\right)}W^{\prime \prime }-g_{33}^{\left(l\right)}X^{\prime \prime }-\mu_{33}^{\left(l\right)}Y^{\prime \prime }+ik\left(q_{15}^{\left(l\right)}+q_{31}^{\left(l\right)}\right)U^{\prime }+likz^{-1}q_{31}^{\left(l\right)}U\\ +lz^{-1}\left(q_{33}^{\left(l\right)}W^{\prime }-g_{33}^{\left(l\right)}X^{\prime }-\mu_{33}^{\left(l\right)}Y^{\prime }\right)-k^{2}q_{15}^{\left(l\right)}W+k^{2}g_{11}^{\left(l\right)}X+k^{2}\mu_{11}^{\left(l\right)}Y]\pi \left(z\right)\\ +\left(\delta (z-0)-\delta (z-h)\right){\left(\frac{z}{h}\right)}^{l}\left(q_{33}^{\left(l\right)}W^{\prime }-g_{33}^{\left(l\right)}X^{\prime }-\mu_{33}^{\left(l\right)}Y^{\prime }+ikq_{31}^{\left(l\right)}U\right)=0 \end{array}$$

where *U* and *W* respectively represent the amplitude of vibration in the *x* and *z* directions. The superscript (’) is the derivative with respect to *z*.

The four amplitudes can be expanded into Legendre orthogonal polynomial series as:

(8) $$U(z)=\sum_{m=0}^{\infty }p_{m}^{1}Q_{m}(z),\;W(z)=\sum_{m=0}^{\infty }p_{m}^{2}Q_{m}(z),\;X(z)=\sum_{m=0}^{\infty }p_{m}^{3}Q_{m}(z),\;Y(z)=\sum_{m=0}^{\infty }p_{m}^{4}Q_{m}(z)$$

where $p_{m}^{\alpha }(\alpha =1,2,3,4)$ are the expansion coefficients, $Q_{m}(r)$ are an orthonormal set of polynomials in the interval [0,*h*].

(9) $$Q_{m}(z)=\sqrt{\frac{2m+1}{h}}P_{m}(\frac{2z-h}{h})$$

where *P_m_* is the Legendre polynomial of order *m*. 

Substituting Equations (8) and (9) into Equation (7), then multiplying both sides of the modified Equation (7) by the complex conjugate $Q_{j}^{*}(z)$ with *j* running from 0 to *M*, integrating over *z* from 0 to *h*, taking advantage of the orthonormality of the polynomial, yields:

(10) k2[Al11j,mAl12j,mAl13j,mAl14j,mAl21j,mAl22j,mAl23j,mAl24j,mAl31j,mAl32j,mAl33j,mAl34j,mAl41j,mAl42j,mAl43j,mAl44j,m]{pm1pm2pm3pm4}+k[Bl11j,mBl12j,mBl13j,mBl14j,mBl21j,mBl22j,mBl23j,mBl24j,mBl31j,mBl32j,mBl33j,mBl34j,mBl41j,mBl42j,mBl43j,mBl44j,m]{pm1pm2pm3pm4}+[Cl11j,mCl12j,mCl13j,mCl14j,mCl21j,mCl22j,mCl23j,mCl24j,mCl31j,mCl32j,mCl33j,mCl34j,mCl41j,mCl42j,mCl43j,mCl44j,m]{pm1pm2pm3pm4}=−ω2[Mlmj0000Mlmj0000000000]{pm1pm2pm3pm4}

or is abbreviated as

(11) $$k^{2}A\cdot p+k^{1}B\cdot p+C\cdot p=-\omega^{2}M\cdot p$$

where **A**, **B**, **C** and **M** are matrices of order 4(*M* + 1)·(*M* + 1), $p={\left[p_{m}^{1}\;p_{m}^{2}\;p_{m}^{3}\;p_{m}^{4}\right]}^{T}$, the elements of the matrices are as following,

Al11j,m=−1hlC11(l)β(m,l,0,j)Al22j,m=−1hlC55(l)β(m,l,0,j)Al23j,m=−1hle15(l)β(m,l,0,j)Al24j,m=−1hlq15(l)β(m,l,0,j)Al32j,m=−1hle15(l)β(m,l,0,j)Al33j,m=1hl∈11(l)β(m,l,0,j)Al34j,m=1hlg11(l)β(m,l,0,j)Al42j,m=−1hlq15(l)β(m,l,0,j)Al43j,m=1hlg11(l)β(m,l,0,j)Al44j,m=1hlμ11(l)β(m,l,0,j)Al12j,m=Al21j,m=0Al13j,m=Al31j,m=0Al14j,m=Al41j,m=0;Bl12j,m=1hl{i(C13(l)+C55(l))β(m,l,1,j)+liC55(l)β(m,l−1,0,j)+iC55(l)γ(m,l,0,j)},Bl13j,m=1hl{i(e15(l)+e31(l))β(m,l,1,j)+lie15(l)β(m,l−1,0,j)+ie15(l)γ(m,l,0,j)}Bl14j,m=1hl{i(q15(l)+q31(l))β(m,l,1,j)+liq15(l)β(m,l−1,0,j)+iq15(l)γ(m,l,0,j)}Bl21j,m=1hl{i(C13(l)+C55(l))β(m,l,1,j)+liC13(l)β(m,l−1,0,j)+iC13(l)γ(m,l,0,j)}Bl31j,m=1hl{i(e15(l)+e31(l))β(m,l,1,j)+lie31(l)β(m,l−1,0,j)+ie31(l)γ(m,l,0,j)}Bl41j,m=1hl{i(q15(l)+q31(l))β(m,l,1,j)+liq31(l)β(m,l−1,0,j)+iq31(l)γ(m,l,0,j)},Bl11j,m=Bl22j,m=Bl33j,m=Bl44j,m=0Bl23j,m=Bl32j,m=0Bl24j,m=Bl42j,m=0Bl34j,m=Bl43j,m=0;Cl11j,m=1hl{C55(l)β(m,l,2,j)+lC55(l)β(m,l−1,1,j)+C55(l)γ(m,l,1,j)}Cl22j,m=1hl{C33(l)β(m,l,2,j)+lC33(l)β(m,l−1,1,j)+C33(l)γ(m,l,1,j)}Cl23j,m=1hl{e33(l)β(m,l,2,j)+le33(l)β(m,l−1,1,j)+e33(l)γ(m,l,1,j)}Cl24j,m=1hl{q33(l)β(m,l,2,j)+lq33(l)β(m,l−1,1,j)+q33(l)γ(m,l,1,j)},Cl32j,m=1hl{e33(l)β(m,l,2,j)+le33(l)β(m,l−1,1,j)+e33(l)γ(m,l,1,j)}Cl33j,m=1hl{−∈33(l)β(m,l,2,j)−l∈33(l)β(m,l−1,1,j)−∈33(l)γ(m,l,1,j)}Cl34j,m=1hl{−g33(l)β(m,l,2,j)−lg33(l)β(m,l−1,1,j)−g33(l)γ(m,l,1,j)}Cl42j,m=1hl{q33(l)β(m,l,2,j)+lq33(l)β(m,l−1,1,j)+q33(l)γ(m,l,1,j)}Cl43j,m=1hl{−g33(l)β(m,l,2,j)−lg33(l)β(m,l−1,1,j)−g33(l)γ(m,l,1,j)}Cl44j,m=1hl{−μ33(l)β(m,l,2,j)−lμ33(l)β(m,l−1,1,j)−μ33(l)γ(m,l,1,j)},Cl12j,m=Cl21j,m=0Cl13j,m=Cl31j,m=0Cl14j,m=Cl41j,m=0Mlmj=1hlρ(l)β(m,l,0,j);with β(m,l,n,j)=∫0hQj*(z)zl∂nQm(z)∂zndz,γ(m,l,n,j)=∫0hQj*(z)zl∂π(z)∂z∂nQm(z)∂zndz.

The objective is to find wave numbers *k* that satisfy the Equation (11). It is simple and useful for propagating wave, by specifying real *k* and then solving for *ω*. But if interest is the evanescent wave, the approach is useless because *k* is complex and the solving of Equation (11) involves a multivariable search. In order to overcome this difficulty, we develop a new solution procedure as shown below. 

We introduce two new vectors:

(12) $$q=k\cdot p,\;N=-\omega^{2}M.$$

Substitution Equation (12) into Equation (11), and then multiplying both sides of the modified Equation (11) by inverse matrix $A^{-1}$, yields

(13) $$A^{-1}(N-C)p-(A^{-1}B)q=k\cdot q.$$

Combining Equation (13) and the above vector q=k⋅p, we obtain

(14) $$\left[\begin{array}{cc} Z & I_{4(M+1)}\\ A^{-1}\left(N-C\right) & {-A}^{-1}B \end{array}\right]\left[\begin{array}{c} p\\ q \end{array}\right]=k\left[\begin{array}{c} p\\ q \end{array}\right].$$

where **I** is the identity matrix and **Z** is a zero matrix.

If we define $R={\left[p\;q\right]}^{T}$, then Equation (14) can be written as

(15) $$\left[\begin{array}{cc} Z & I_{4(M+1)}\\ A^{-1}\left(N-C\right) & {-A}^{-1}B \end{array}\right]R=kR.$$

Up to this stage, the problem is reduced to a typical eigenvalue problem, which can be easily solved using an eigensolver routine that yields the complex eigenvalues *k*. All the developments performed in this paper were implemented in Mathematica software (version 8.0, Wolfram company, Champaign, IL, USA). The calculation technique in the short-circuit case is similar to that which is used in the open-circuit case. The deduction process is not shown to save space.

## 3. Numerical Results and Discussion

Based on the previous formulations, the computer program in terms of the presented method has been written using Mathematica software to calculate the dispersion and phase velocity curves for the FGPPM plate composed of CoFe_2_O_4_ (top) and Ba_2_TiO_3_ (bottom), *h* = 1 mm. The material parameters are from literature [26] and are listed in Table 1. 

We use the Voigt-type model, as described in the literature [27], to calculate the effective material property of the FGPPM plate:

(16) $$F(z)=F_{B}V_{B}(z)+F_{C}V_{C}(z),\;V_{B}(z)+V_{C}(z)=1$$

where *F_B_* and *F_C_* respectively represent the material property of the Ba_2_TiO_3_ and CoFe_2_O_4_ materials, and *V_B_* and *V_C_* are volume fraction.

Equation (16) can be rewritten as

(17) $$F(z)=F_{B}+(F_{C}-F_{B})V_{C}(z)$$

Similar to Equation (5), *V_C_*(*z*) can be expressed as a power expansion, Here we consider four different gradient fields, $V_{C}(z)={\left(z/h\right)}^{n}$, *n* = 1, 2 and 3, namely linear, quadratic and cubic graded fields, and sinusoidal graded field $V_{C}(z)=\sin(0.5\pi z/h)$.

### 3.1. Approach Validation and Convergence of the Problem

**Figure 2:**
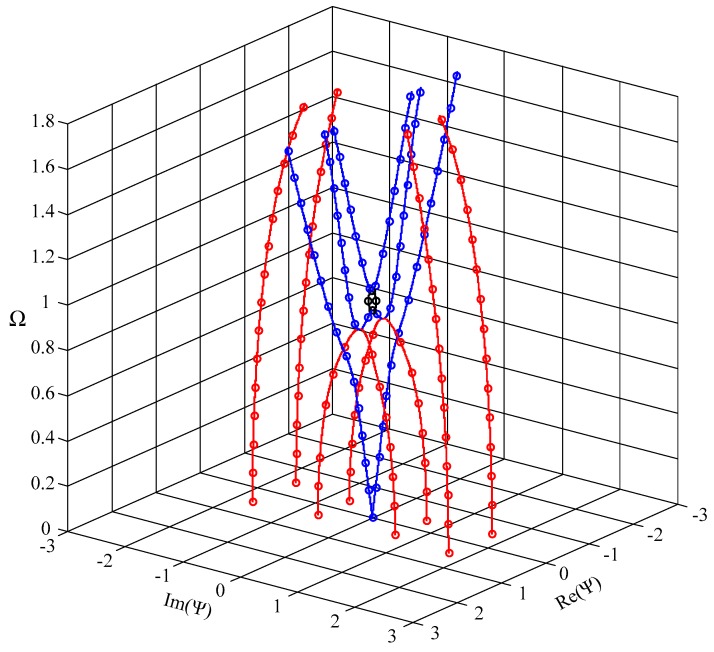
Dispersion curves of Lamb wave in a steel plate; hollow dots-our results, solid lines-literature results from the spectral collocation method; real branch in blue, purely imaginary branch in black, complex branch in red.

To check the validity and the efficiency of our approach, we make a comparison between our results and the literature results. Because there is no investigation on the evanescent waves in FGPPM so far, we compute the full spectrum of Lamb wave in a steel plate and make a comparison with the available results in literature [17] from a spectral collocation method. The calculating parameters are *ρ* = 7932 kg/m^3^, *C*_11_ = 281.757 GPa, *C*_12_ = 113.161 GPa, *C*_44_ = 84.298 GPa, and *h* = 10 mm. The non-dimensional frequency and wave number are defined as $\Omega =\left(\omega h\sqrt{\rho /C44}\right)/\pi$, Ψ=kh/π, respectively. The resulting dispersion curves are given in Figure 2. It clearly shows that the numerical results obtained by the present polynomial approach agree well with those obtained by the spectral collocation method, which validates our approach and program. 

**Figure 3:**
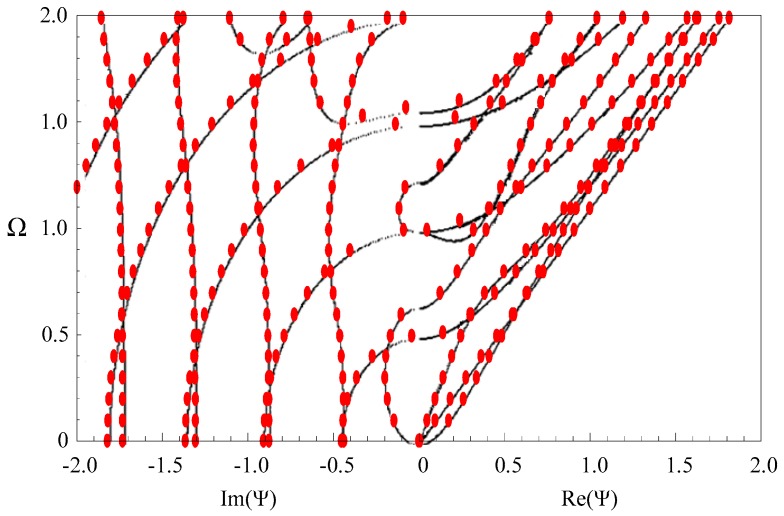
Dispersion curves of Lamb wave in a PZT-4 plate; red dots—our results, black dotted lines—literature results from the reverberation-ray matrix method.

The material in the above verification example is isotropic. We calculate the dispersion curves of Lamb wave in an orthotropic plate and make a comparison with the available results in literature [28] from the reverberation-ray matrix method, which serves as a further validation of our approach. The material is PZT-4, and the material parameters are listed in Table 2. Figure 3 shows the obtained frequency spectra. Here again, the agreement is quite good between our results and those from the reverberation ray matrix method.

**Figure 4:**
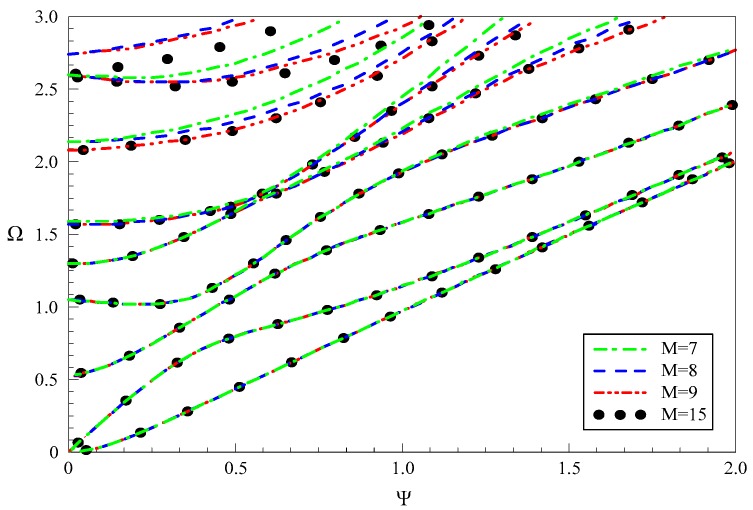
Dispersion curves of propagating Lamb-like wave in a linear functionally graded piezoelectric-piezomagnetic material (FGPPM) plate with various “*M*”.

Then we discuss the convergence of the present polynomial approach. We present dispersion curves of propagating Lamb-like wave in a linear FGPPM plate with electric and magnetic open circuit and *h* = 1 mm, when the truncation order *M* takes 7, 8, 9 and 15, respectively, as shown in Figure 4. It can be seen that more and more order modes converge as *M* increases. When *M* = 7, the first three modes are convergent. The first four when *M* = 8, and the first seven when *M* = 9. So, we can think that at least the first (*M* − 1)/2 modes are convergent. Similarly, this can be concluded for the purely imaginary modes, and we don’t present the dispersion curves of purely imaginary branches for saving space. For evanescent Lamb-like waves, we tabulate the results in Table 3 since graph is not convenient for comparison. These numerical results also show that the complex solutions are convergent as *M* increases. When *M* = 10 and *M* = 11, the first three modes are convergent. The first four when *M* = 12, the first five when *M* = 13, and the first six when *M* = 14. Obviously, the real solution is easier to converge than the complex one. From these results, good convergence of the present approach can be observed. We take *M* = 30 in this paper.

### 3.2. Full Dispersion Curves of Lamb Wave

**Figure 5:**
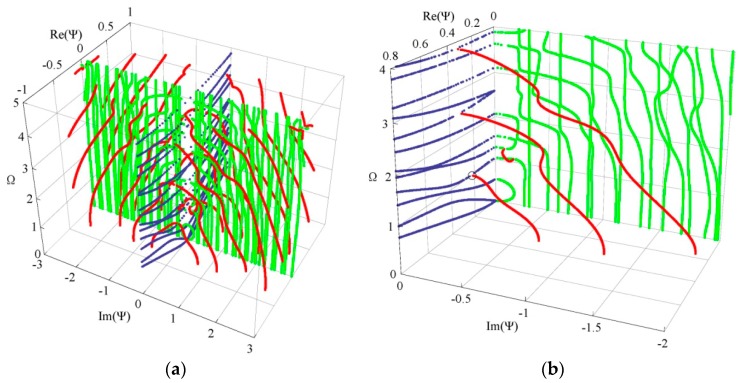
3D dispersion curves of Lamb wave: (**a**) four quadrants, (**b**) one quadrant; blue—real solutions, green—imaginary solutions, red—complex solutions.

Propagating waves have received a lot of attention, and here we put the emphasis on evanescent waves. We plot the full dispersion curves in 3D frequency-complex wave number space for a clearer visualization of the solutions and a better understanding of the nature of the modes, when necessary, with a different color for clarity. Figure 5a plots the full dispersion curves of Lamb wave for a linear FGPPM plate with electric and magnetic open circuit. Since the eigenvalues are computed for one *ω* at a time, the dispersion curves are constructed of unconnected dots and the points near the cut-off frequencies become sparse. We can observe that purely real and purely imaginary solutions appear in pairs of opposite signs and the complex ones appear in quadruples of complex conjugates and opposite signs. Purely real wave numbers correspond to the propagating wave, and purely imaginary and complex wave numbers correspond to the evanescent wave. For a given frequency, a certain small number of real branches exist together with an infinite number of complex and purely imaginary branches (mostly imaginary with few complexes in the given range). For clarity, Figure 5b shows one quadrant dispersion curves in a small range. For complex branches, most of them start from 0 frequency and end at the minima of the purely real branches. Occasionally, one connecting two purely imaginary branches appears. The real part of the complex branches is usually small. For purely imaginary branches, most of them start from 0 frequency and end at cut-off frequencies with increasing frequency, and some with small wave numbers start from one cut-off frequency and terminate the other one.

**Figure 6:**
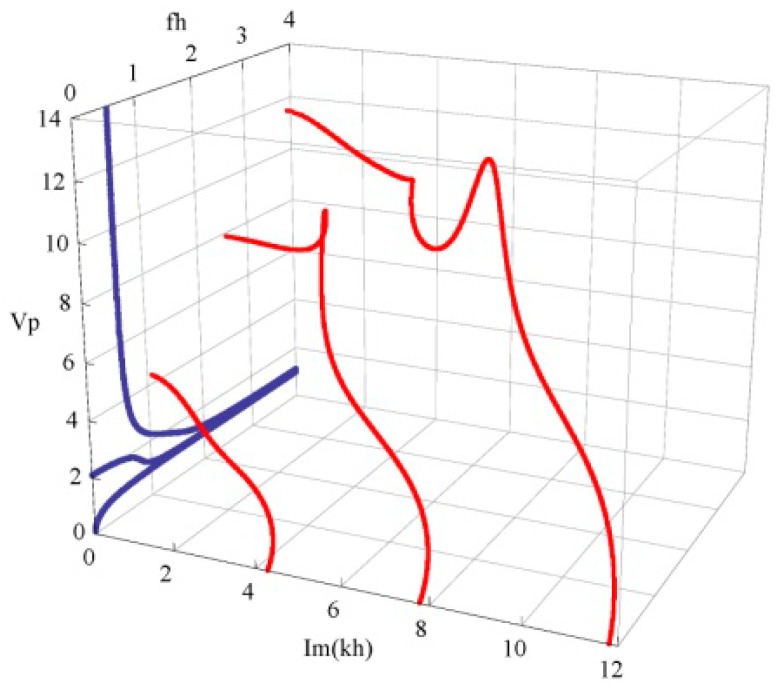
The phase velocity dispersion and attenuation curves; propagating wave in blue, evanescent wave in red.

Figure 6 shows the phase velocity dispersion and attenuation curves of the first three propagating and complex branches. The dimensionless phase velocity and frequency and attenuation are defined by Vp=ω/(Re(k)⋅C55/ρ), $\mathrm{fh}=\omega h/(2\pi \sqrt{C_{55}/\rho })$ and Im(kh). We can find from these curves that the phase velocity of a propagating mode is decreased and gradually tends to a steady value with increasing frequency, but the velocity of an evanescent mode becomes bigger as well as the attenuation decreases. At high frequency, the evanescent mode has a very small attenuation, and its phase velocity is noticeably bigger than that of a propagating mode. For example, at *fh* = 2–3, the phase velocity of the second evanescent mode is about 8, but that of the propagating mode is below 2. Also, the wave dispersion is quite weak in this frequency range.

### 3.3. Influences of Graded Field on Dispersion Curves and the Electromechanical Coupling Factor

**Figure 7:**
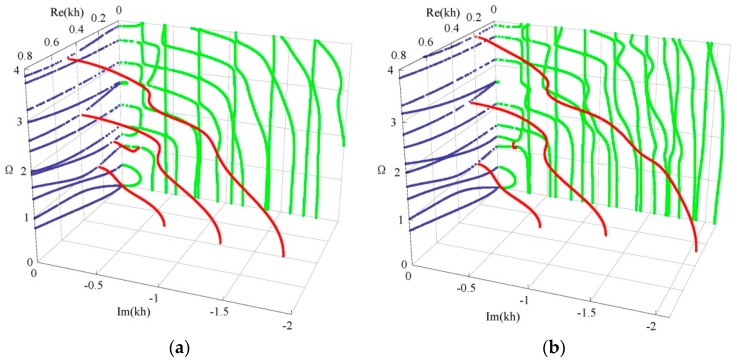
3D dispersion curves of Lamb wave: (**a**) cubic graded field, (**b**) sinusoidal graded field. blue—real branches, green—purely imaginary branches, red—complex branches.

**Figure 8:**
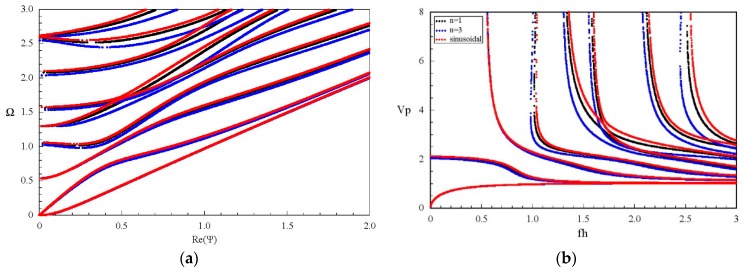
Dispersion curves of propagating Lamb wave for FGPPM plates with different graded fields; (**a**) Frequency spectra; (**b**) Phase velocity spectra.

**Figure 9:**
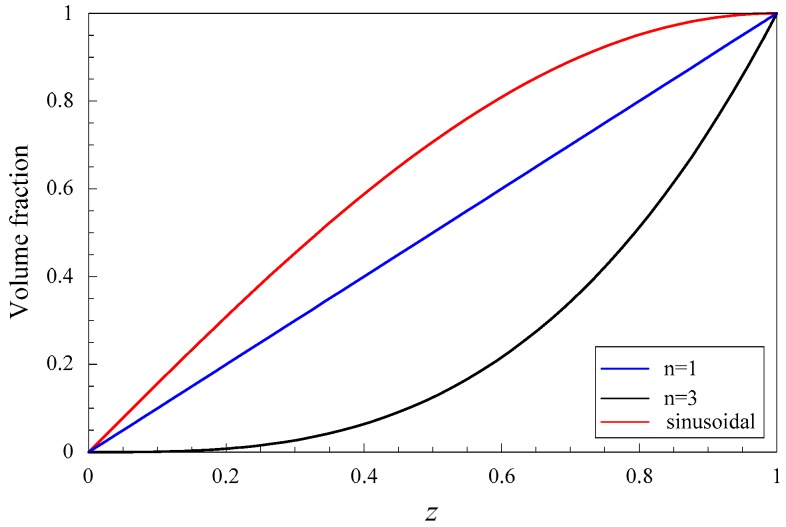
Variation curves of the three graded functions.

Considering two graded shapes, cubic and sinusoidal graded fields. Figure 7 shows their dispersion curves of Lamb wave. The results show that the effect of the graded field on dispersion characteristics of Lamb wave is significant, including the propagating modes and evanescent modes. Comparison between Figure 7 and Figure 5b, we can notice that the imaginary part of the complex branches for the sinusoidal graded case, at Ω = 0 plane, is bigger than that for the linear and cubic cases. Interestingly, for the cubic graded cases, the complex branch connecting two purely imaginary branches disappears and turns into a different one connecting a purely imaginary branch and a real branch. For clarity, Figure 8 shows the frequency spectra and phase velocity spectra of Lamb propagating wave for the three graded fields. Obviously, the effect of the graded field is little on the low mode, but becomes significant with increasing the mode order and wave number. The phase velocity for the sinusoidal graded field is bigger than that for the linear graded field, while the linear bigger than the cubic. The reason lies in that the different graded fields result in different material volume distributions, and the wave velocity depends on the material properties. Figure 9 gives the variation curves of the three gradient fields in the *z* direction. The CoFe_2_O_4_ content for the sinusoidal graded field is the highest, and the wave velocity of Ba_2_TiO_3_ is slower than that of CoFe_2_O_4_. 

The magneto-electromechanical coupling factor *K*^2^ is an important parameter for designing acoustic wave devices. A high magneto-electromechanical coupling factor is expected in engineering applications. It is defined as [29]

(18) $$K^{2}=\frac{2\left|Voc-Vsc\right|}{Voc}$$

where *Voc* and *Vsc* are the phase velocities for the electric and magnetic open circuit and short circuit, respectively. 

**Figure 10:**
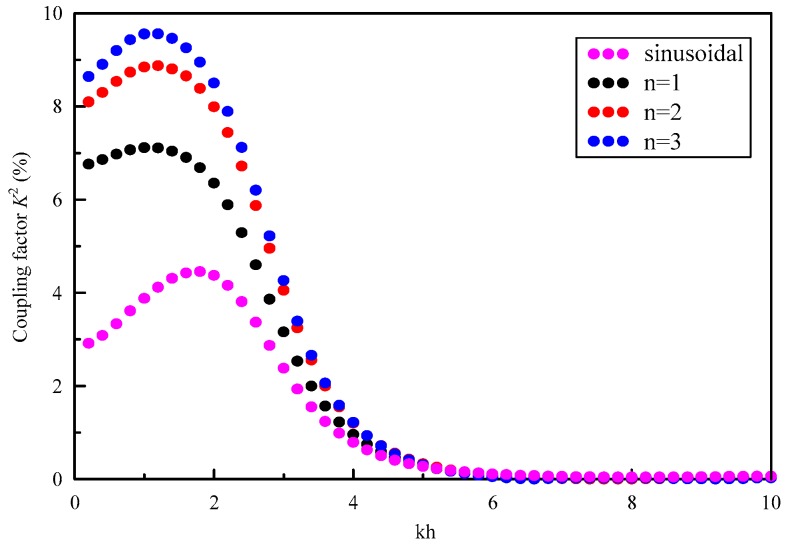
Effect of graded field on magneto-electromechanical coupling factor for S0 mode.

To illustrate the effect of graded field on the *K*^2^, we calculate the *K*^2^ for S0 modes of four different FGPPM plates, as shown in Figure 10. We can find that the *K*^2^ reaches a maximum at a certain wave number and tend to the same little value with increasing wave number, which implies the influence of the graded field on the energy propagation of Lamb wave in high-frequency zone is insignificant. It reaches a maximum from 4.4% for the sinusoidal graded field to 9.5% for the cubic graded field. They are located near *kh* = 2 and *kh* = 1.5 respectively. The *K*^2^ for the cubic graded field is always bigger than that of the other three graded cases. Also the maximum of *K*^2^ shifts to the smaller wave number when the graded power exponent is increasing. 

### 3.4. Wave Structure Analysis

**Figure 11:**
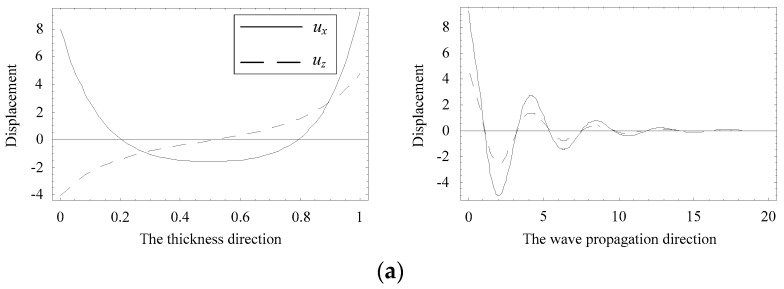

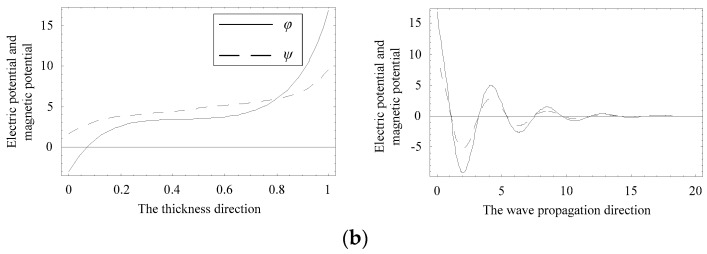
Distributions of the physical quantities when Ω = 1.01115, Ψ = 0.23216 − 0.04612i. (**a**) displacement distribution, (**b**) electric potential and magnetic potential distribution.

**Figure 12:**
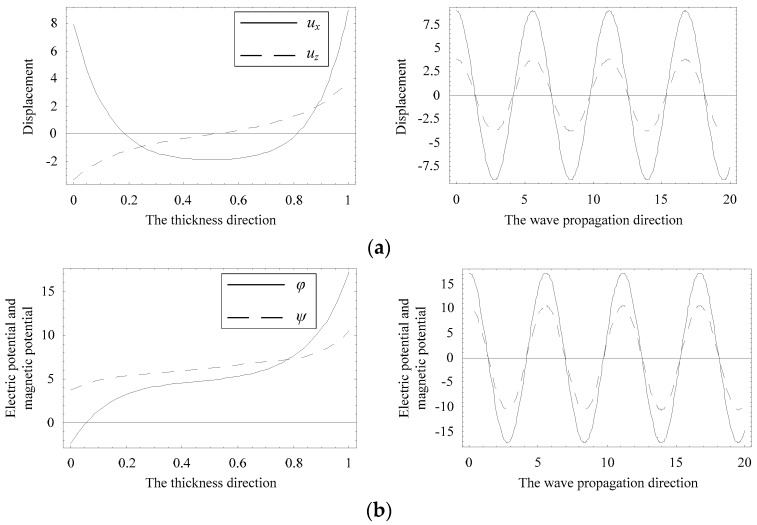
Distributions of the physical quantities when Ω = 1.01911, Ψ = 0.17938. (**a**) displacement distribution, (**b**) electric potential and magnetic potential distribution.

The distributions of displacement and electric potential and magnetic potential fields can be obtained according to Equations (4) and (8). Considering a special position where the complex branch firstly collapses onto the real branch at about Ω = 1.0, as marked with a circle in Figure 5b. Figure 11 and Figure 12 present the distributions of the physical quantities in the *z* and *x* directions when Ω = 1.01115, Ψ = 0.23216 − 0.04612i, and Ω = 1.01911, Ψ = 0.17938, respectively. As seen in these figures, the real branch propagates without any attenuation, and the complex branch exhibits an oscillatory distribution and propagates a very long distance, about a few tens of thicknesses of the plate. The displacement *u_z_* and electric potential and magnetic potential distributions change along the *z* direction in a nearly anti-symmetric manner. The displacement *u_x_* exhibits a nearly symmetric manner. The distribution of displacement *u_z_* of the complex branch is very similar to that of the real branch, implying the evanescent wave mode converts into the propagating wave mode.

### 3.5. Merits of the Presented Method

Based on the above calculation of wave propagation in a FGPPM plate, we can summarize the following advantages of the presented method, which makes the method attractive.

(1) The complex mathematical issue is reduced to solve an eigenvalue problem, which is capable of accurately determining all the real, imaginary and complex solutions of a transcendental dispersion equation.
(2) The conventional approaches (root-finding routines or finite element simulations) require an iterative search procedure or a far greater coding effort, to find complex roots. The present method can avoid tedious iterative two-variable search and is simple to program. It needs to take a larger polynomial order to obtain solutions of the higher modes, which will cause more computer memory and long time.
(3) The method is easy to implement and can be extended to complex structures such as multilayered or curved structures.

## 4. Conclusions

This paper presents an analytic method based on polynomial expansions for the determination of the full dispersion spectrum of the guided waves in the FGPPM plate. The correctness of the present method is verified via numerical comparison with available reference results. For the first time, the complete 3D dispersion curves of Lamb wave in a FGPPM plate are illustrated in a wide frequency range. The characteristics of the Lamb waves including the propagating and evanescent modes in various FGPPM plates are investigated. The emphasis on evanescent waves makes this work relevant for applications in the nondestructive evaluation of material or structural properties. Based on the above numerical results, some interesting conclusions can be drawn:

(1) Superior to the conventional methods that necessitate an iterative search procedure to solve the complex roots of a dispersion equation, the presented analytic method can transform the set of differential equations for the acoustic waves into an eigenvalue problem in the form AX = *k*X to find the complex solutions.
(2) Complex branches of the Lamb wave usually collapse onto the extremum of the real branches. They exhibit both local vibration and local propagation, and some can propagate a quite long distance (more than ten times of the plate thickness). They will turn into the propagating modes with increasing frequency.
(3) Some evanescent modes have a noticeably higher phase velocity than the propagating modes. The phase velocity of the low order evanescent modes is more than four times larger than that of the propagating modes. Also, the wave dispersion of the evanescent mode is quite weak in a certain frequency range.
(4) The magneto-electromechanical coupling factor of the guided wave in a FGPPM plate may be improved by adjusting the graded field. The coupling factor reaches a maximum from 4.4% for the sinusoidal graded field to 9.5% for the cubic graded field. The maximum of the magneto-electromechanical coupling factor for the S0 mode shifts to lower frequencies with increasing the gradient index.

## Figures and Tables

**Table 1:** Material parameters of two piezoelectric-piezomagnetic materials.

**Materials**	**Property**								
	***C*_11_**	***C*_12_**	***C*_13_**	***C*_22_**	***C*_23_**	***C*_33_**	***C*_44_**	***C*_55_**	***C*_66_**
Ba_2_TiO_3_	166	77	78	166	78	162	43	43	44.6
CoFe_2_O_4_	286	173	170	286	170	269	45.3	45.3	46.5
	**Property**								
	***e*_15_**	***e*_24_**	***e*_31_**	***e*_32_**	***e*_33_**	***∈*_11_**	***∈*_22_**	***∈*_33_**	***ρ***
Ba_2_TiO_3_	11.6	11.6	−4.4	−4.3	18.6	196	201	28	5.8
CoFe_2_O_4_	0	0	0	0	0	0.8	0.8	0.93	5.3
	**Property**								
	***q*_15_**	***q*_24_**	***q*_31_**	***q*_32_**	***q*_33_**	***μ*_11_**	***μ*_22_**	***μ*_33_**	
Ba_2_TiO_3_	0	0	0	0	0	5	5	10	
CoFe_2_O_4_	550	550	580.3	580.3	699.7	−590	−590	157	

Units: *C_ij_* (10^9^ N/m^2^), *∈_ij_* (10^−10^ F/m), *e_ij_* (C/m^2^), *q_ij_* (N/Am), *μ_ij_* (10^−6^ Ns^2^/C^2^), *ρ* (10^3^ kg/m^3^).

**Table 2:** Material parameters.

**Material**	**Property**								
	***C*_11_**	***C*_12_**	***C*_13_**	***C*_22_**	***C*_23_**	***C*_33_**	***C*_44_**	***C*_55_**	***C*_66_**
PZT-4	139	78	74	139	74	115	25.6	25.6	30.5
	**Property**								
	***e*_15_**	***e*_24_**	***e*_31_**	***e*_32_**	***e*_33_**	***∈*_11_**	***∈*_22_**	***∈*_33_**	***ρ***
PZT-4	12.7	12.7	−5.2	−5.2	15.1	65	65	56	7.5

Units: *C_ij_* (10^9^ N/m^2^), *∈_ij_* (10^−10^ F/m), *e_ij_* (C/m^2^), *ρ* (10^3^ kg/m^3^).

**Table 3:** Convergence of complex wave numbers of the first six modes (Ω = 0.1).

*M*	Mode					
	1	2	3	4	5	6
10	0.31308 +0.67653i	0.32055 +1.23591i ™	0.16593 +1.87713i ™	0.33292 +2.58928i ™	0.34536 +3.14395i ™	0.24222 +3.74226i ™
11	0.31308 +0.67653i	0.32055 +1.23591i ™	0.16593 +1.87713i ™	0.33184 +2.59041i	0.34722 +3.14407i ™	0.24481 +3.73460i ™
12	0.31308 +0.67653i	0.32055 +1.23591i ™	0.16593 +1.87713i ™	0.33063 +2.59061i	0.35203 +3.14418i ™	0.25047 +3.72524i
13	0.31308 +0.67653i	0.32055 +1.23591i ™	0.16593 +1.87713i ™	0.33063 +2.59061i	0.35211 +3.14436i ™	0.25504 +3.72496i ™
14	0.31308 +0.67653i	0.32055 +1.23591i ™	0.16593 +1.87713i ™	0.33063 +2.59061i	0.35211 +3.14436i ™	0.25561 +3.72471i ™
20	0.31308 +0.67653i	0.32055 +1.23591i ™	0.16593 +1.87713i ™	0.33063 +2.59061i	0.35211 +3.14436i ™	0.25561 +3.72471i ™
